# Characterization and determination of holin protein of *Streptococcus suis *bacteriophage SMP in heterologous host

**DOI:** 10.1186/1743-422X-9-70

**Published:** 2012-03-22

**Authors:** Yibo Shi, Yaxian Yan, Wenhui Ji, Bin Du, Xiangpeng Meng, Hengan Wang, Jianhe Sun

**Affiliations:** 1 Department of Animal Science, School of Agriculture and Biology, Shanghai Jiao Tong University, Shanghai Key Laboratory of Veterinary Biotechnology, 800 Dongchuan Road, Shanghai, People's Republic of China

**Keywords:** *Streptococcus suis*, Bacteriophage, Holin, Lysin

## Abstract

**Background:**

Holins are a group of phage-encoded membrane proteins that control access of phage-encoded endolysins to the peptidoglycan, and thereby trigger the lysis process at a precise time point as the 'lysis clock'. SMP is an isolated and characterized *Streptococcus suis *lytic phage. The aims of this study were to determine the holin gene, *HolSMP*, in the genome of SMP, and characterized the function of holin, HolSMP, in phage infection.

**Results:**

*HolSMP *was predicted to encode a small membrane protein with three hydrophobic transmembrane helices. During SMP infections, *HolSMP *was transcribed as a late gene and HolSMP accumulated harmlessly in the cell membrane before host cell lysis. Expression of *HolSMP *in *Escherichia coli *induced an increase in cytoplasmic membrane permeability, an inhibition of host cell growth and significant cell lysis in the presence of LySMP, the endolysin of phage SMP. HolSMP was prematurely triggered by the addition of energy poison to the medium. *HolSMP *complemented the defective λ *S *allele in a non-suppressing *Escherichia coli *strain to produce phage plaques.

**Conclusions:**

Our results suggest that HolSMP is the holin protein of phage SMP and a two-step lysis system exists in SMP.

## Background

Holin-lysin lysis systems typically exist in the double-stranded DNA bacteriophages for termination of their growth cycle and release of viral progeny through host cell lysis. By accumulating and forming lesions in the cytoplasmic membrane, holins control access of phage-encoded endolysins to the peptidoglycan and thereby trigger lysis of the host cell at a precise time point. This process determines the length of the infection cycle and is known as the 'lysis clock'.

It is known that holins do not share sequence similarity, although they do have some common characteristics. Firstly, most holins are encoded by the gene adjacent to the endolysin gene. Secondly, at least one hydrophobic transmembrane domain (TMD) occurs in all holins. Thirdly, holins have a highly charged, hydrophilic, C-terminal domain. By identifying these characteristics, it is possible to predict putative holins. Holins can be grouped into three classes by topology. Class I holins, such as bacteriophage λ S protein [1] and *Staphylococcus aureus *phage P68 hol15 protein [2], generally have more than 95 residues and form three TMDs. Class II holins, such as the S protein from lambdoid phage 21 [3] and the Hol3626 protein from *Clostridium perfringens *bacteriophage Ф3626 [4], are smaller (65 to 95 residues) and form two TMDs. Class III holins, such as the holin of ФCP39O and ФCP26F [5], just have one TMD in the central region of the molecule. The schedulings of lysis time by some holin genes are specified by the dual-start model. In the dual-start model, the holin gene is an open reading frame that encodes two proteins, holin and antiholin, with opposing functions that are responsible for the accurate timing of the endolysin release [6,7]. For example, the prototype class I holin gene, the *S *gene of bacteriophage λ encodes not only the effector holin, S105, but also an inhibitor, S107, with a Met_1_-Lys_2_-Met_3_... extension at the terminus. An sdi (site-directed initiation) structure near the 5' end of the *S *gene controls translational initiations from the two initiator codons and determines the ratio of holin to antiholin.

Holins from bacteriophages infecting Gram-negative bacteria have been widely studied, especially bacteriophage λ [8], bacteriophage T4 [9] and bacteriophage PRD [10,11]. In Gram-positive bacteria, several studies on holins have been conducted in phage infecting host cells such as *Staphylococcus aureus *[2,12], *Lactococcus lactis *[13,14], *Lactobacillus fermentum *[15], *Streptococcus thermophilus *[16], *Streptococcus pneumoniae *[17,18], *Listeria monocytogenes *[19] and *Bacillus cereus *[20]. However, no studies on holins from bacteriophages infecting *Streptococcus suis *(*S. suis*) have been reported

*S. suis *is an important pathogen of pigs causing arthritis, endocarditis, meningitis, pneumonia and septicemia [21]. Thirty-five serotypes (types 1 to 34 and 1/2) based on capsular antigens are currently known. Serotype 2 is considered the most virulent and prevalent type in diseased pigs in China. SMP, an *S. suis *serotype 2 lytic phage, was isolated and characterized in our previous work. Analysis of the complete genomic sequence (GenBank: EF116926) revealed the presence of a putative holin-lysin lysis system [22], thus providing further evidence that this is the universal mechanism to schedule host lysis for dsDNA phages. Phage-encoded lysins which could degrade peptidoglycan of Gram-positive bacteria exogenously have a bright future as potential therapeutic agents [23]. The extracellular lytic activities of LySMP, the putative endolysin of SMP, on *S suis *and its biofilm have been tested and confirmed [24,25], and HolSMP, the putative holin of SMP, was also showed synergistic antibacterial activity against *S. suis *with LySMP in our recent work (data not published). However, the exact structure and function of HolSMP remained to be investigated. The inability to isolate bacteria lysogenic for SMP limits the functional analysis of the holin gene. Fortunately, holin-dependent induction of membrane lesions is nonspecific, and this enables testing of holins encoded by bacteriophages infecting Gram-positive bacteria in *Escherichia coli *(*E. coli*) [26]. Therefore, in this study, the putative holin, HolSMP, was functionally analyzed in *E. coli*.

## Results and discussion

### Computational predictions and analyses of HolSMP

**Figure 1:**
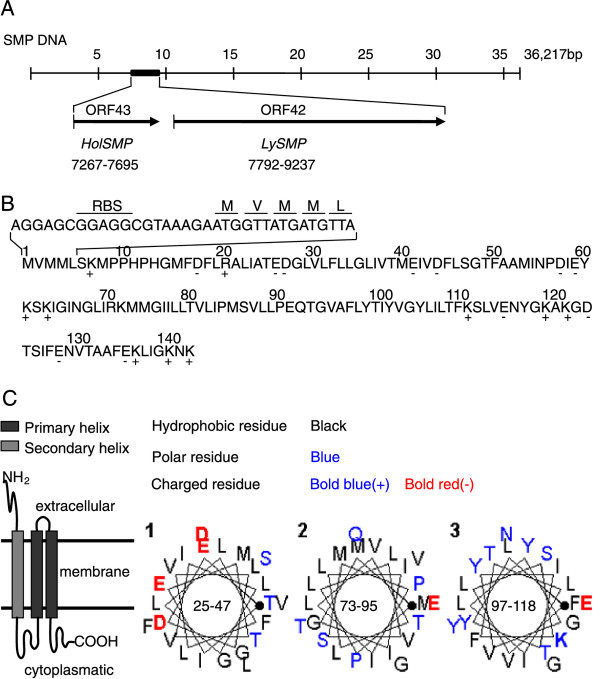
**ORF43 from the complete sequence of SMP was analyzed**. (**A**) Location of *LySMP *(ORF42) and *HolSMP *(ORF43), which are likely to comprise the putative holin-lysin lysis system of SMP. (**B**) The primary sequence of the *HolSMP *product. Charged residues are indicated below the sequence. (**C**) Secondary structure of HolSMP predicted by SOSUI.

The complete 36,126-bp sequence of phage SMP has 48 open reading frames (ORF). ORF42, designated *LySMP*, encodes the putative endolysin. ORF43, which is 429 bp long and upstream of the *lySMP *gene, is predicted to encode a putative holin protein, designated *HolSMP *(Figure 1A). There is a 96-bp sequence located between *HolSMP *and *LySMP*. A ribosome-binding site (RBS) was identified upstream of the first start codon (Figure 1B). HolSMP consists of 142 amino acids (15.7 kDa) and shows 88% similarity to the sequence of a putative holin of *Streptococcus *phage MM1 (accession number NP 150180), and exhibits the characteristics of phage-holin_4 superfamily. The results of PredictProtein, TmHMM and SOSUI analysis suggested that HolSMP is a membrane protein with traits typical of a holin. HolSMP has three putative hydrophobic TMDs and its N-terminus extends into the periplasm (Figure 1C). HolSMP is rich in methionine residues. Five of them, Met_1_, Met_3_, Met_4_, Met_8 _and Met_15_, are located upstream of TMD_1_. Met_40 _is within TMD_1_. Met_53 _is located between TMD_1 _and TMD_2_, and the three other Met codons, Met_73_, Met_74 _and Met_85_, are all within TMD_2 _(Figure 1C). HolSMP has a hydrophilic C-terminus with several charged amino acids, whereas the N-terminus has three positively charged amino acids and only one negatively charged residue. HolSMP shares structural characteristics of holins, and HolSMP should be assigned to class I.

**Figure 2:**
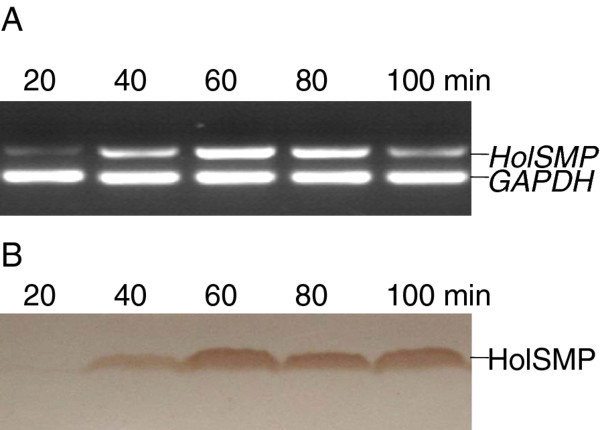
**Detection of transcription and expression of gene *HolSMP *in *S. suis *after phage infection**. (**A**) Reverse transcription PCR was performed to evaluate *HolSMP *transcript levels. The quantities of *HolSMP *transcripts in each sample were compared, while the house-keeping gene, *GAPDH*, was employed as a reference for normalization of samples. (**B**) Western blotting was performed on the membrane fraction extracted from phage infected host cells.

**Figure 3:**
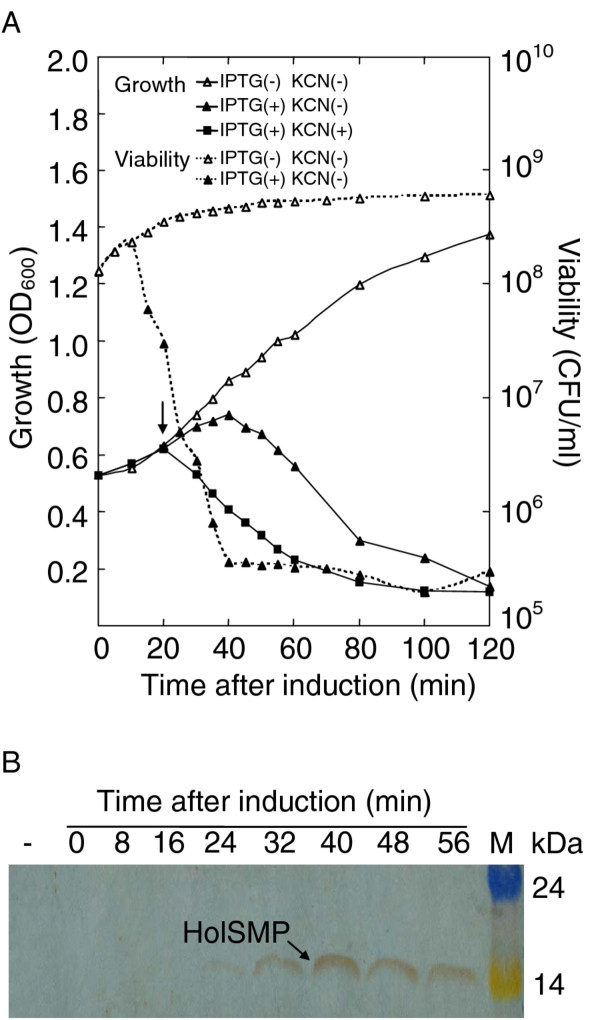
**HolSMP is toxic to *E. coli *and cyanide triggers the lethal potential of HolSMP**. (**A**) The growth and viability assays of BL21(DE3)pLysS harboring plasmid pEXH1 were performed after addition of IPTG. At the time indicated by the arrow, KCN was added to the culture. Non-induced BL21(DE3)pLysS (pEXH1) was also monitored as control. (**B**) Western blotting was performed on total cellular protein of IPTG induced BL21(DE3)pLysS (pEXH1) to detect expression of HolSMP. Cultures of BL21(DE3)pLysS (pEX) were collected at 56 min after induction as a negative control (-). A size marker is shown in the last lane.

The dual-start model is still to be explored in holins of SMP although it is found in most other bacteriophages. Although HolSMP is rich in Met residues at the C-terminus, the Lys residue is absent at the N-terminus (Met_1_-Val_2_-Met_3_-Met_4_...), and no sdi structure has been found near the 5' end of *HolSMP*. The western blot results of HolSMP expression in both homologous and heterologous hosts, showed a single detectable band at the expected position (Figures 2B and 3B), but whether HolSMP is the unique product of *HolSMP *should be investigated further by methods such as the toeprinting assay. The present data do not support the dual-start model for phage SMP. In bacteriophge λ S105 protein, a unique but crucial cysteine is shown to cause the formation of disulfide-linked dimmers under oxidative conditions, which suggested a model of holin to form lesion in the membrane [27], but no cysteine is occupied by HolSMP, which might imply a different lesion-formation model of holin from the one of S105 protein.

### Expression of HolSMP in *S.suis *during phage SMP infection

The one-step growth curve of SMP has been studied previously and showed a latent period of at least 20 min and a rise period of 120 min [22]. To determine the transcription of *HolSMP *in SMP-infected *S. suis*, samples were collected at 0, 5, 10, 15, 20, 40, 60, 80, 100 and 120 min after infection. Total RNA was extracted from the samples and was reverse transcribed into cDNA, and fragments of *HolSMP *(473 bp) and *glyceraldehydes-3-phosphate dehydrogenase *(*GAPDH*, 223 bp) gene were amplified by PCR. The electrophoresis result showed that the *GAPDH *gene of host cells could be detected in all samples, while the *HolSMP *gene could only be detected after 20 min. Moreover, the transcript level of *HolSMP *kept rising rapidly before 60 min (Figure 2A) and then started to decline. Real-time quantitative PCR relatively quantified the accumulation of *HolSMP *mRNA (Table 1). The results showed that *HolSMP *transcripts were undetectable before 15 min. A sharp increase appeared before 60 min. The numbers of *HolSMP *transcripts at 20, 40 and 60 min were 10, 523 and 1, 722 times greater than at 15 min, respectively. Decreased *HolSMP *transcript levels were observed after 60 min and lasted to the end of the experiment at 120 min. Further, to determine the product of *HolSMP*, host cells were collected at 20, 40, 60, 80 and 100 min after infection and separated from cultures by centrifugation. It was notable that samples collected at 60 and 80 min were viscous, indicating the action of a holin-lysin lysis system and the release of progeny phages. The membrane fraction was extracted from each sample and examined by western blotting. One single band corresponding to HolSMP (15.7 kDa) appeared at the expected position of each lane except for the sample collected at 20 min (Figure 2B), suggesting that HolSMP is located in the membrane of phage-infected host cells. Accumulation of HolSMP in the membrane was found before 60 min. With a reduction of *HolSMP *transcripts and destruction of host cells, the amount of HolSMP in the membrane also started to reduce. All of the above results show that *HolSMP *is a late gene. Transcription and expression of *HolSMP *does not occur during the early stage of infection but is highly up-regulated when progeny phages are released.

**Table 1:** Relative quantitation of *HolSMP *using the comparative C_T _method

Time (min)	*HolSMP *Average C_T_	*GAPDH *Average C_T_	ΔC_T _*HolSMP- GAPDH*^a^	ΔΔC_T _ΔC_T_-ΔC_T_, _0 min_^b^	*HolSMP *Rel. to 0 min^c^
0	30.61 ± 0.09	16.64 ± 0.03	13.97 ± 0.09	0.00 ± 0.09	1.0 (0.9-1.1)
5	30.66 ± 0.03	16.83 ± 0.03	13.83 ± 0.04	-0.14 ± 0.04	1.1 (1.07-1.13)
10	29.60 ± 0.05	18.40 ± 0.03	11.20 ± 0.06	-2.77 ± 0.06	6.8 (6.5-7.1)
15	24.54 ± 0.03	16.30 ± 0.05	8.24 ± 0.06	-5.73 ± 0.06	53.1 (50.9-55.3)
20	21.27 ± 0.02	16.38 ± 0.04	4.89 ± 0.04	-9.08 ± 0.04	541.2 (526.4-556.4)
40	17.69 ± 0.03	18.49 ± 0.03	-0.80 ± 0.04	-14.77 ± 0.04	27939.1 (27175.1-28724.6)
60	16.10 ± 0.02	18.61 ± 0.05	-2.51 ± 0.05	-16.48 ± 0.05	91405.9 (88292.3-94629.3)
80	16.42 ± 0.03	18.55 ± 0.02	-2.12 ± 0.04	-16.09 ± 0.04	69754.6 (67847.1-71715.6)
100	15.26 ± 0.07	16.56 ± 0.04	-1.30 ± 0.08	-15.27 ± 0.08	39511.9 (37380.6-41764.8)
120	17.94 ± 0.03	18.68 ± 0.01	-0.74 ± 0.03	-14.71 ± 0.03	26801.0 (26249.5-27364.2)

a. The ΔC_T _value is determined by subtracting the average *GAPDH *C_T _value from the average *HolSMP *C_T _value. The standard deviation of the difference is calculated from the standard deviation of the *HolSMP *and *GAPDH *values.

b. The calculation of ΔΔC_T _involves subtraction by the ΔC_T _calibrator value. This is subtraction of an arbitrary constant, so the standard deviation of ΔΔC_T _is the same as the standard deviation of the ΔC_T _value.

c. The range given for *HolSMP *relative to 0 min is determined by evaluation the expression: 2^-ΔΔCT ^with ΔΔC_T _+ s and ΔΔC_T _- s, where s = the standard deviation of the ΔΔC_T _value.

### Expression of HolSMP in *E. coli*

*E. coli *is a convenient host for the investigation of holin proteins from phage that infect Gram-positive bacteria [26]. Therefore, the functional identification of putative holin protein HolSMP was performed in BL21(DE3)pLysS (pEXH1) strains. The plasmid pEXH1, containing the *HolSMP *gene sequence, was constructed. BL21(DE3)pLysS (pEXH1) was created and growth of transformants was monitored after induction with β-D-thiogalactopyranoside (IPTG) (Figure 3A). Growth inhibition of induced cells occurred from 25 to 40 min and was followed by cell lysis with the OD_600 _value reducing from 0.85 to 0.24. Toxicity of HolSMP to BL21(DE3)pLysS cells was further proven by the viability assay of induced cells. It was showed that the number of viable cells began to decrease at 10 min and a three-log-unit drop was observed (Figure 3A). Moreover, as is characteristic of all holins, HolSMP could be triggered prematurely by the addition of energy poison, potassium cyanide (KCN, 10 mM) (Figure 3A). To determine the kinetics of HolSMP expression, total cellular protein samples were prepared every 8 min, and the accumulation of HolSMP was determined by western blotting. The results showed that a 16 kDa species, consistent with the predicted mass of HolSMP, was detected in samples taken after 24 min, but not before (Figure 3B). The level of HolSMP protein increased from 24 min to 40 min and then declined.

HolSMP accumulated in and damaged the membrane of expressing cells. In order to confirm the subcellular location of HolSMP in cells, cultures of BL21(DE3)pLysS (pEXH1) were collected 40 min after induction to isolate total cellular protein, cytoplasmic protein and membrane protein samples. As the negative control, protein fractions were also prepared from BL21(DE3)pLysS (pEX). Western blots revealed that the dark brown bands indicating HolSMP protein appeared in total cellular protein preparations and the membrane fraction of BL21(DE3)pLysS (pEXH1), but not in the cytoplasmic fraction or in the HolSMP-negative subcellular samples. This suggested that HolSMP accumulates in the membrane of *E. coli*, consistent with *S. suis*.

**Figure 4:**
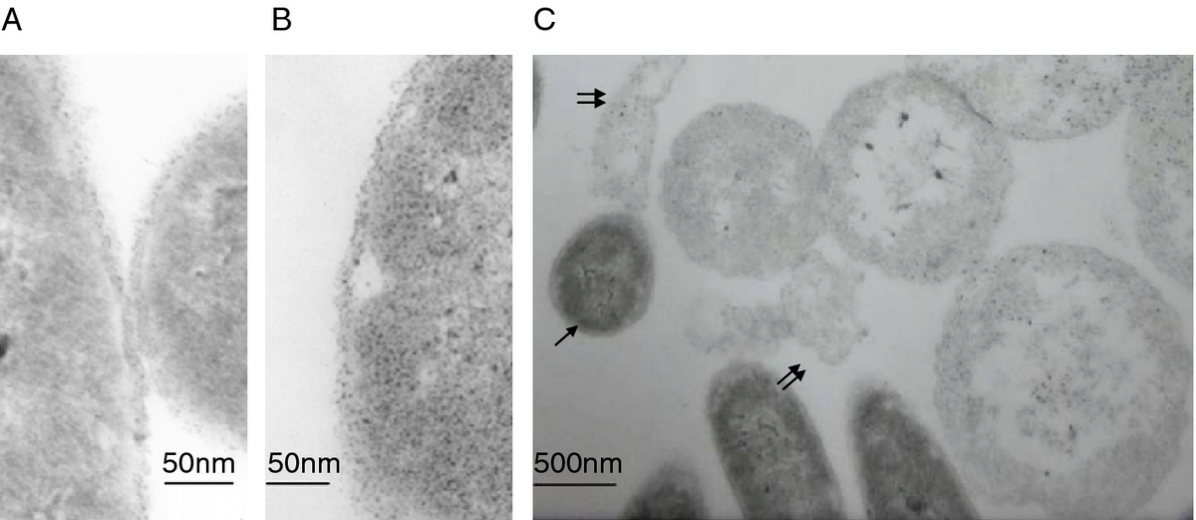
**Representative views of morphological changes of BL21(DE3)pLysS (pEXH1)**. Compared to uninduced cells (**A**), the membrane of induced cells (**B**) separated from the cell wall. BL21(DE3)pLysS (pEXH1) became quite large in size (**C**), with an incomplete cell wall which eventually became fragmented. The single arrow indicates a cell of normal size, while double arrows indicate debris of broken cells.

Changes in cell morphology during HolSMP expression after induction were explored by observing cells harboring plasmid pEXH1 with phase contrast microscopy. BL21(DE3) cells induced with IPTG appeared translucent and non-refractile, with a normal shape. The expected changes in transparency and refraction of cells were also observed in the cell wall after induction of the holin genes of phage λ, Ф29 and pneumococcal bacteriophage EJ-1 [17]. It has been reported that holin, even in the absence of lysin, is lethal for the host cell since it causes increased cytoplasmic membrane permeability, collapse of the membrane potential, inhibition of respiration and defective active transport. BL21(DE3)pLysS (pEXH1) cells also appeared translucent and non-refractile after induction. Furthermore, BL21(DE3)pLysS (pEXH1) cells were round and somewhat larger than normal cells. Therefore, ultrathin sections of induced BL21(DE3)pLysS (pEXH1) cells were prepared and examined by electron microscopy. A subtle separation of the cytoplasmic membrane from the cell wall was observed in induced cells when the cells were still of normal size (Figure 4A and 4B). A dramatic expansion of cells and an indistinct cell wall could be observed about 1 h after induction (Figure 4C). The phenomenon was consistent with the phase contrast microscopy. BL21(DE3)pLysS harbors the pLysS plasmid, which carries the gene encoding T7 lysozyme. Therefore, it was deduced that the expression of HolSMP in BL21(DE3)pLysS compromised the cytoplasmic membrane, leading to the release of T7 lysozyme and further damage to the cell wall, resulting in the observed changes in shape and size. Our observations are indirect evidence that accumulation of HolSMP leads to lesion formation in the cytoplasmic membrane, through which some proteins might pass and execute their function.

### Determination of HolSMP as a holin protein

**Figure 5:**
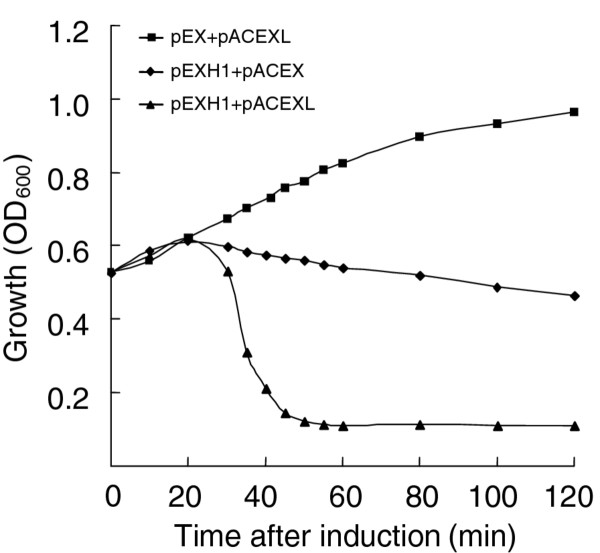
**Co-expression of *HolSMP *and *LySMP *results rapid lysis of BL21(DE3) host cells**. The growth of BL21(DE3) cells were monitored by absorbance determination after cotransformation with pEX + pACEXL (expressing only LySMP), pEXH1 + pACEX (expressing HolSMP), and pEXH1 + pACEXL (expressing both HolSMP and LySMP), respectively.

LySMP, the SMP endolysin encoded by gene *LySMP*, cannot cause cell lysis without the assistance of holin. To further identify the physiological role of HolSMP as a holin protein for the release of endolysin, we co-expressed *HolSMP *with *LySMP *in BL21(DE3). The growth of clones co-transformed with pACEXL and pEXH1 were monitored after induction with IPTG (Figure 5). The results showed that cells co-transformed with pEX and pACEXL, expressing only LySMP, did not cause any host cells lysis. Co-transformation with pEXH1 and pACEX, expressing HolSMP, started to inhibit the growth of host cells as early as 10 min followed by a slow decrease in absorbance 20 min after induction. However, the coexpression of *HolSMP *and *LySMP *resulted in an abrupt decrease in absorbance from 20 min. These results suggested that HolSMP does not significantly lyse cells, but might permit the release of LySMP, which results in the abrupt decrease in absorbance.

**Figure 6:**
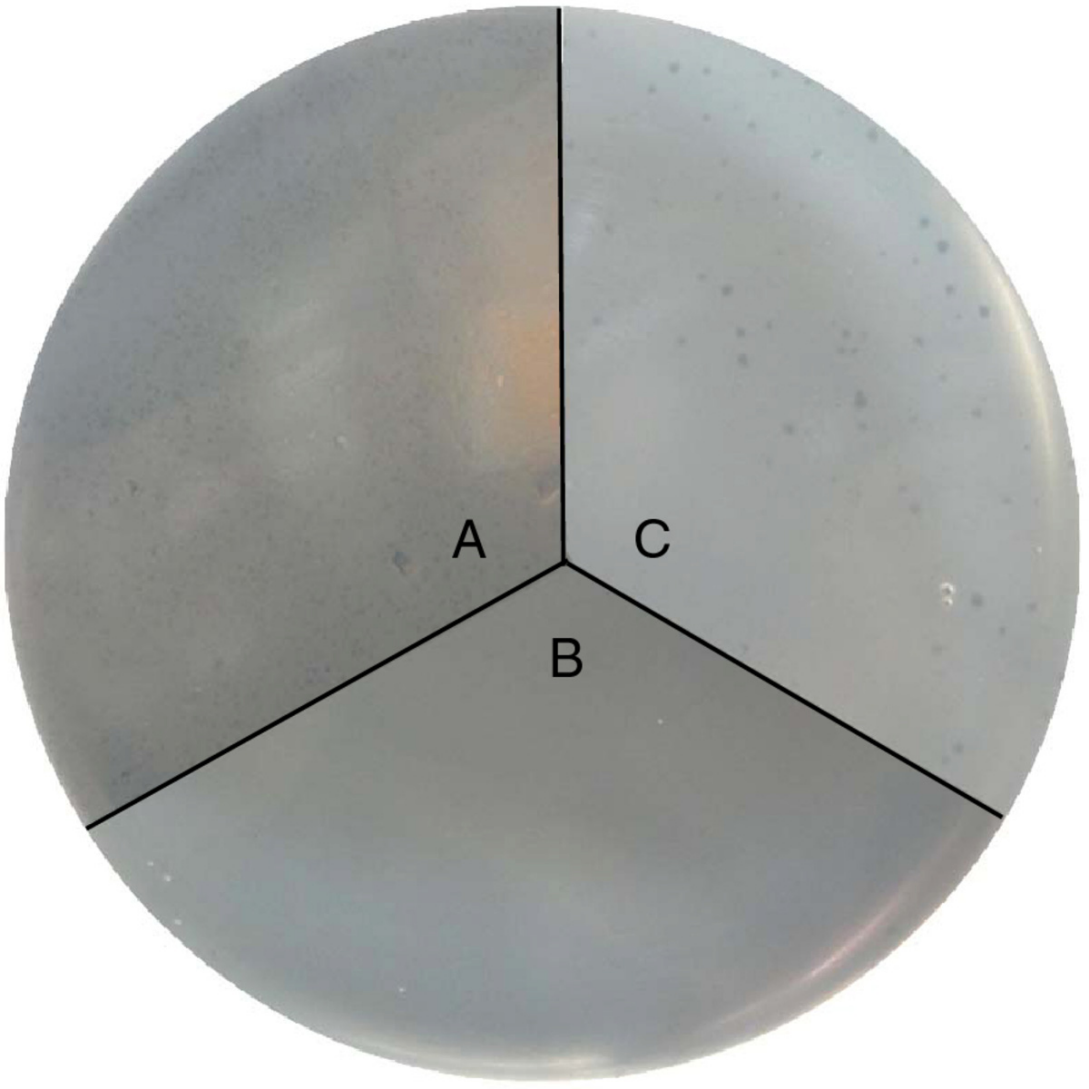
**HolSMP functions as a holin to complement S-negative phage λ *c*I857 *Sam*7**. (**A**) Host cell lysis by phage λ *c*I857 *Sam*7 requires a permissive host, featuring *supF*, such as VCS257. (**B**) Plaques will not be produced on plates with nonermissive host BL2(DE3)pLysS. (**C**) With the expression of sub-lethal levels of HolSMP, large and clear plaques were observed on plate with host BL2(DE3)pLysS (pEXH1).

HolSMP complements an S-negative lysis-defective λ phage mutant. It is known that holins are essential for endolysin R of the λ phage to display lytic activity. Phage λ *c*I857 *Sam*7 carries an amber mutation in the *S *gene and, consequently, cannot trigger lysis of the infected host cells unless a suppressing *E. coli *strain is used. To further document the role of HolSMP, we carried out complementation tests using the non-suppressing strain BL21(DE3)pLysS (pEXH1) and phage λ *c*I857 *Sam*7. BL21(DE3)pLysS (pEXH1), infected by phage λ *c*I857 *Sam*7, was added to soft agar containing IPTG. Because the high efficiency of expression of the pET system may lead to hyper-expression of HolSMP and cell toxicity, the IPTG concentration was reduced to 0.1 mM. This induces expression of HolSMP at sublethal levels. Plaques were observed when *HolSMP *was induced. No plaques formed on the plates of BL21(DE3)pLysS harboring plasmid pET-32a(+) used as a control plasmid for pEXH1. The defective bacteriophage λ *c*I857 *Sam*7 can form plaques when grown on the suppressing strain VCS257. Moreover, plaques formed by phage λ *c*I857 *Sam*7 on the complementation plates were larger and clearer than the *supF *host VCS257 plates (Figure 6). The result of the complementation test indicated that HolSMP is able to function as a holin protein and complement an S-negative lysis-defective λ phage mutant.

## Conclusions

From these results, protein HolSMP has been identified for the first holin of *S. suis *bacteriophage. The holin gene, *HolSMP*, located upstream of the endolysin gene is transcribed, expressed in *S. suis *after infection by phage SMP. HolSMP, a putative member of class I holins, accumulates on membrane of *S. suis*. At present, an *S. suis *strain lysogenic for SMP has not been isolated, and it is not easy to obtain *HolSMP*-defective SMP mutants. However, biological evidence for the holin-like character of HolSMP was obtained in a Gram-negative background. The HolSMP product caused cellular death, and changes in cell morphology could be accounted for by lesions in the membrane. By forming lesions in the cytoplasmic membrane, HolSMP permitted T7 lysozyme in BL21(DE3)pLysS, LySMP in BL21(DE3), and R in phage λ-infected cells to escape through the membrane and damage the cell wall. HolSMP shows the same host non-specificity and membrane energy sensitivity as other holins [17,18]. Thereby, the holin-lysin lysis system of SMP was also determined in this study. The HolSMP is able to trigger activity of the LySMP and release viral progeny through host cell lysis. Our further biochemical investigations will shed light of the mechanism of HolSMP action and the application of HolSMP in biopharmacy

## Methods

### Bacterial strains, phages, plasmids and growth conditions

Bacterial strains, phages, and plasmids used in this study are listed in Table 2. *S. suis *SS2-H was grown in Todd-Hewitt broth (THB) or agar medium, supplemented with 2% (v/v) newborn bovine serum, at 37°C. Preparation of bacteriophage SMP was carried out as described previously [24]. Briefly, SMP was propagated on SS2-H by the double-layer agar plate method and eluted with phage buffer containing 100 mM NaCl, 8 mM MgSO_4_, 50 mM Tris, pH 7.5 and 0.1‰(w/v) gelatin. SMP was separated from the host by filtration. *E. coli *strains were cultured in standard Luria-Bertani medium (LB) supplemented with ampicillin (100 μg/ml) (LB-Ap) or chloramphenicol (30 μg/ml) (LB-Cm) or both (LB-Ap-Cm), as appropriate. In BL21(DE3)pLysS, pLysS-encoded T7 lysozyme inhibits T7 RNA polymerase to reduce basal expression of target gene and is also able to degrade the peptidoglycan layer of the cell wall when membrane lesions exist. The suppressing strain VCS257 was cultured in LB supplemented with 10 mM MgSO_4 _and 0.2% (w/v) maltose for infection by phage λ *c*I857 *Sam*7. Liquid cultures were incubated overnight at 37°C with shaking at 200 rpm.

**Table 2:** Bacterial strains, phages and plasmids used in this study

Strains, phages or plasmids	Genotype and relevant features	Source
Strains DH5α	*E. coli *F^- ^endA1 glnV44 thi-1 recA1 relA1 gyrA96 deoR nupG Φ80d*lac*ZΔM15 Δ(*lacZYA- argF*)U169, hsdR17(r_K_^- ^m_K_^+^), λ-	Tiangen
BL21(DE3)	*E. coli F^-^ompT hsdS(r_B_^-^m_B_^-^) gal dcm (DE3)*	Tiangen
BL21(DE3) plysS	*E. coli *F^-^*ompT hsdS(r_B_^-^m_B_^-^) gal dcm *(DE3) pLysS (Cm^R^)	Tiangen
VCS 257	DP50 *sup*F[*supE44 supF58 hsd53*(r_B _m_B_)*dap D8lacY1 glnV44 *Δ(*gal-uvrB*)*47 tyrT58 gyrA29 tonA53 *Δ(*thyA57*)]	Stratagene
*SS2-H *Phages	*Streptococcus suis*, serotype 2	Lab stock
SMP	Wild type phage of *S. suis*	Lab stock
λ *c*I857 *Sam*7	cIts857, Sam7,*Lac *promoter for expression of cloned genes, phage of *E. coli*	Stratagene
Plasmids		
pET-32a(+)	Expression vector containing hybrid T7-*lacPO *promoter, Φ10 ribosome binding site, and *lacI *gene (pBR322 derivative), ampicillin resistance	Novagen
pACYC184	Low copynumber cloning vector; p15A *ori*; Tc^+^; Cm^+^	Lab stock
pEXH1	Derivative of pET-32a(+); whole ORF of *HolSMP *gene, *HolSMP(429)*, inserted behind RBS of pET-32a(+)	This study
pEXL	Derivative of pET-32a(+); *LySMP *fragment inserted behind RBS of pET-32a(+)	This study
pEX	Derivative of pET-32a(+); pEXH1 (Δ *HolSMP*)	This study
pACEXL	*Hin*d III-*Sph*I fragment of pEXL subcloned into *Hin*d III-*Sph*I sites of pACYC184	This study
pACEX	*Hin*d III-*Sph*I fragment of pEX subcloned into *Hin*d III-*Sph*I sites of pACYC184	This study

### Computational analyses

DNA and protein sequence homology alignments were performed using BLAST tools on NCBI. TMHMM http://www.cbs.dtu.dk/services/TMHMM/, SOSUI http://bp.nuap.nagoya-u.ac.jp/sosui/ and PredictProtein servers http://www.predictprotein.org were used to predict the transmembrane helices in HolSMP.

### DNA manipulation and plasmid construction

DNA was isolated from SMP as described for phage λ [28]. The *HolSMP *and *LySMP *genes were amplified by PCR from purified phage genomic DNA using primers designed listed in Table 3. The amplified genes were then cloned into prokaryotic expression vectors using two restriction sites incorporated in the PCR primers. The ligation product was transformed into competent *E. coli *DH5α. Recombinant plasmids were extracted from transformants and sequenced. Plasmids were retransformed into competent *E. coli *BL21(DE3) or BL21(DE3)pLysS under selection. It should be noted that there are no tags coding sequences in the recombinant plasmids for which original physiological functions of HolSMP and LySMP remained

**Table 3:** Primers used in this study^a^

Primers	Sequences
EXH1	AACTTTAAGAAGGAGATATACATatggttatgatgtta
EXH2	ACGCTCTAGAAATAATTTTGTTTAACTTTAAGAAGGAG
EXH3	CGCGGATCCctttgattagtttcatttat
EXH4	CGCGGATCCcttatcttaaactggttttt
EXH5	TTAACTTTAAGAAGGAGGAAATCatgacaatcaacatt
SYB34	AACTTTAAGAAGGAGATATACATatggttatgtgatta
SYB35	ttccgatgtcagtgctt
SYB36	ttggctttgccgtagtt
GAPDH1	actatcggcttggtaatcccagaat
GAPDH2	atgaaccgaatgagatacctacga

a. New restriction sites and RBS are indicated by underline and shadow, respectively. Lowercase letters represent sequences corresponding to that of *HolSMP*.

To construct plasmid pEXH1, *HolSMP *was amplified with oligonucleotides EXH1 and EXH3 to generate a PCR fragment (HolSMP-1) containing the RBS of plasmid pET-32a(+). PCR using fragment HolSMP-1 as the template was performed to generate a final 495 bp PCR fragment (HolSMP-2) with primer EXH2 and the common reverse primer EXH3. HolSMP-2 was cloned into the *Xba*I and *BamH*I sites of pET-32a(+) to give plasmid pEXH1. Plasmid pEXL, harboring the *LySMP *gene, was constructed in the same as was plasmid pEXH1, using primer pairs EXH5/EXH4 and EXH2/EXH4.

The negative control plasmid pEX was constructed using the same strategy as for pEXH1, using primer SYB34 to introduce a stop codon at the beginning of *HolSMP*. The expression vectors pACEXL and pACEX were constructed by subcloning the *Hin*dIII-*Sph*I fragment of pEXL (containing the T7 promoter, RBS, *Lac *operon and lysine gene) or pEX (containing T7 promoter, RBS, *Lac *operon and MCS) into *Hin*dIII-*Sph*I digested pACYC184.

### RNA extraction from SMP-infected host cells

To identify the transcription of the *HolSMP *gene in SMP-infected *S. suis*, exponentially growing SS2-H cells (about 10^9^) were infected with phage SMP (10^9^-10^10 ^plaque forming units/ml) at a multiplicity of infection of at least 10. A sample, containing 10^8 ^host cells, was collected prior to addition of phage and immediately centrifuged at 13,000 × *g *for 1 min to pellet cells. The rest of the reaction was incubated at 37°C for 15 min and centrifuged at 13,000 × *g *for 1 min. The cell pellet was re-suspended gently with THB and incubated at 37°C with shaking at 150 rpm. Samples, containing 10^8 ^cells, were collected as described above at 5, 10, 15, 20, 40, 60, 80, 100 and 120 min after infection. Cell pellets were snap frozen in liquid nitrogen as soon as the supernatant was discarded, and stored at -20°C until RNA extraction. Total RNA of all samples was extracted simultaneously with the RNA extraction kit (Omega). Contaminating DNA was removed by digestion. Downstream cDNA synthesis was performed when DNA from SMP and host cells could not be detected by PCR.

### Reverse transcription PCR

The transcript levels of *HolSMP *were determined visually by reverse transcription PCR. MMLV reverse transcriptase (25 U) and random primers (TakaRa) were used for cDNA synthesis. The *S. suis *housekeeping gene *GAPDH *was employed as a reference for normalization of samples.

Two pairs of primers, EXH1/EXH3 and GAPDH1/GAPDH2 (Table 3), were used to amplify *HolSMP *and *GAPDH*, respectively. PCR was carried out in a final volume of 25 μl, containing 2 μl cDNA (1:5 dilution), 0.4 μM of each primer, and 12.5 μl 2 × PCR mix (Dongsheng Biotech). Amplification was performed for 28 cycles with annealing temperatures of 57.5°C and 60°C for *HolSMP *and *GAPDH*, respectively. The volume of each *HolSMP *PCR product loaded for electrophoresis was adjusted based on the corresponding *GAPDH *fragments (223 bp). Gels were visualized with an image analysis system after electrophoresis. Then, bands corresponding to the *HolSMP *gene (473 bp) in each lane were compared.

### Real-time quantitative PCR

Further relative quantification of transcript levels of *HolSMP *was performed by real-time quantitative PCR. Primers SYB35 and SYB36 were designed using primer 5.0 software to amplify 116 bp of *HolSMP *(Table 3). The *S. suis *housekeeping gene *GAPDH *was employed as a reference for normalization of samples. PCR was carried out with a PTC-200 PCR instrument (Bio-Red, Hercules, CA) and MJ option Monitor analysis system. PCR was carried out in a final volume of 50 μl, containing 2 μl cDNA (1:5 dilution), 0.4 μM of each primer, and 1 × SYBR premix EX taq II (Takara). Amplification was performed over 40 cycles of 5 s at 95°C, 30 s at the annealing temperature (57.5°C for *HolSMP *and 60°C for *GAPDH*), and 10 s at 72°C. The reaction products were then cooled to 50°C and subjected to a post-PCR melting cycle by increasing the temperature by 0.2°C every 10 s, up to 95°C. The comparative C_T _method was used to analyze the relative transcription levels of *HolSMP *after infection.

### Membrane protein extraction from SMP-infected host cells

To identify the expression of *HolSMP *in SMP-infected *S. suis*, exponentially growing SS2-H cells were infected with phage SMP as described above. Samples, containing 10^11 ^host cells, were collected as described above at 20, 40, 60, 80 and 100 min after infection. Cell pellets were frozen in liquid nitrogen immediately until required for extraction of membrane proteins. To prepare membrane fractions, harvested cells were suspended in 5 ml ice-cold lysis buffer (300 mM NaCl, 50 mM sodium phosphate, pH8.0), and sonicated on ice at 200 W for 50 cycles of 3 s on and 20 s off. The cell fragments were collected by centrifugation at 13,000 × *g *for 1 min. This process was repeated until cells were completely lysed. The collected supernatant was ultracentrifuged at 100,000 × *g *for 1 h at 4°C to pellet membrane fragments. Each pellet was solubilized with 5 ml ME buffer (1%Triton X-100, 10% glycerine, 0.5 M NaCl, 35 mM MgCl_2_, 220 mM Tris-HCl, pH8.0) and incubated for 12 h on ice with shaking [29]. The insoluble fraction was discarded after ultracentrifugation at 100,000 × *g *for 1 h at 4°C. Note that addition of lysozyme must be avoided.

### Protein expression and viability assays

BL21(DE3)pLysS harboring plasmid pEXH1, designated BL21(DE3)pLysS (pEXH1), was inoculated and cultured to an optical density at 600 nm (OD_600_) of 0.5 ~ 0.6. Protein expression was induced by addition of IPTG to a final concentration of 1 mM and shaking at 30°C at 150 rpm. The growth of clones after induction was monitored by measuring OD_600_. For protein expression analysis, cells in 1 ml cultures were suspended with 100 μl 1 × tricine sample buffer (1 × TSB, 50 mM Tris-HCl [pH 6.8] containing 12% [w/v] glycerol, 4% [w/v] SDS, 2.5% [v/v] mercaptoethanol and 0.01% [w/v] bromophenol blue) and boiled for about 5 min to prepare total cellular protein samples. For viability assays, 20 μl cultures of BL21(DE3)pLysS carrying plasmid pEXH1 was placed on ice at different time points after addition of IPTG. Each sample was serially diluted on ice and 100 μl dilutions were plated in triplicate on LB-Ap. Colonies from three separate experiments were counted after 12 to 16 h of incubation at 37°C.

### Subcellular fractionation

One litre culture of BL21(DE3)pLysS (pEXH1) induced for 40 min by IPTG was harvested by centrifuging at 13,000 × *g *for 3 min at 4°C. To prepare the cytoplasmic fraction, harvested cells were suspended in 5 ml ice cold lysis buffer, sonicated on ice at 400 W for 20 min, (3 s on/20 s off cycles), and ultracentrifuged at 100,000 × *g *for 1 h at 4°C to remove the membrane fraction. Membrane protein preparation from membrane pellets was performed with 5 ml ME buffer as described in membrane protein extraction from SMP-infected host cells. Both cytoplasmic fraction and membrane fraction samples were mixed with 2 × tricine sample buffer and boiled.

### Tricine-SDS-PAGE and western blots

HolSMP was separated by tricine-SDS-PAGE and examined by western blotting. For tricine-SDS-PAGE, the protein samples were resolved on 20% (w/v) polyacrylamide gels as previously described [30]. The gel was stained with Coomassie blue or directly used to transfer proteins onto a nitrocellulose membrane by electroblotting. The antibody against a recombinant protein corresponding to TMD_2_-TMD_3_-C-terminal sequence of HolSMP was raised in mouse in our lab previously. For immunodetection of HolSMP, the antibody against HolSMP (1:1000 dilution) and the goat anti-mouse immunoglobulin conjugated to horseradish peroxidase (1:2500 dilution; Immunology Consultants Laboratory, Inc.) were used as the primary and secondary antibodies, respectively. The western blots were analyzed with DAB colorimetric western blot kit (Rockland).

### Transmission electron microscopy

Culture samples were collected every 5 min in the first hour after the addition of IPTG and centrifuged at 1,160 × *g *for 3 min to pellet cells. The pellets were re-suspended in 2.5% glutaraldehyde in 0.1 M PBS (pH 7.4). Cells were fixed at 4°C for 30 min and centrifuged at 1,160 × *g *for 1 min. Thin sections of the cells were processed and examined at 60,000 × magnification with a Hitachi H-600 transmission electron microscope.

### Co-expression of *HolSMP *and *LySMP *in *E. coli*

To explain the physiological role of HolSMP, *HolSMP *was co-expressed with *LySMP*. The *Hin*dIII-*Sph*I fragment containing *LySMP *from pEXL and a negative control sequence from pEX were inserted into plasmid pACYC184. The resulting plasmids were designated pACEXL and pACEX. The chloramphenicol-resistant plasmid pACYC184 harboring the p15A origin of replication was compatible with colE1 of vector pET-32a(+) [31]. Therefore, the recombinant plasmids pACEXL and pACEX were compatible with pEXH1. The *E. coli *BL21(DE3) strains harboring plasmid combinations pEXH1+pACEXL (harboring both *HolSMP *and *LySMP*), pEXH1+pACEX (harboring *HolSMP *only) and pACEXL+pEX (harboring *LySMP *only) were grown overnight in LB-Ap-Cm. The strains were diluted (1:100) with fresh medium and cultured to an OD_600 _of 0.6. Expression of the genes was induced by addition of IPTG, and the growth of clones was monitored by measuring OD_600_.

### Complementation of λ Sam 7 lysis function

BL21(DE3)pLysS (pEXH1) was inoculated and cultured to an OD_600 _of about 0.5 in LB-Ap. A 200-μl BL21(DE3)pLysS culture was infected at 37°C for 15 min with 10 μl bacteriophage λ *c*I857 *Sam*7 (10^5 ^plaque forming units/ml). The *E. coli *BL21(DE3)pLysS and phage were mixed with 5 ml of soft agar containing 0.1 mM IPTG and 100 μg/ml ampicillin, and quickly poured onto LB-Ap plates. Addition of 0.1 mM IPTG to the soft agar induced expression of *HolSMP *at sub-lethal levels for BL21(DE3)pLysS harboring plasmid pEXH1 unless the R lysin of λ *c*I857 *Sam*7 was also present. The plates were incubated face up at 37°C to encourage the formation of plaques, and the number of plaques was determined after overnight incubation. BL21(DE3)pLysS with plasmid pET-32a(+) and VCS257 were used as controls. Before infection, freshly cultured VCS257 was gently resuspended and diluted to an OD_600 _of 0.5 with sterile 10 mM MgSO_4 _after centrifugation at 500 × *g *for 10 min. Antibiotic was not added to the soft agar or plates to culture VCS257.

## Abbreviations

*E. coli*: *Escherichia coli*; *GAPDH*: *Glyceraldehydes-3-phosphate dehydrogenase *gene; KCN: Potassium cyanide; IPTG: β-D-thiogalactopyranoside; LB: Luria-Bertani medium; LB-Ap: LB supplemented with ampicillin (100 μg/ml); LB-Ap-Cm: LB supplemented with ampicillin (100 μg/ml) and chloramphenicol (30 μg/ml); LB-Cm: LB supplemented with chloramphenicol (30 μg/ml); ORF: Open reading frame; RBS: Ribosome-binding site; sdi: Site-directed initiation; *S. suis*: *Streptococcus suis*; THB: Todd-Hewitt broth; TMD: Transmembrane domain.

## Competing interests

The authors declare that they have no competing interests.

## Authors' contributions

YBS and JHS carried out the design of the study, analyzed the data and drafted the manuscript. WHJ performed the plaque and viability assays. DB participated in the transmission electron microscopy analysis. XPM conceived of the study, participated in its design and coordination and helped to draft the manuscript. YXY and HAW participated in the design of the study. All authors read and approved the final manuscript.
